# “That is why I have trust”: unpacking what ‘trust’ means to participants in international genetic research in Pakistan and Denmark

**DOI:** 10.1007/s11019-017-9795-9

**Published:** 2017-09-05

**Authors:** Zainab Sheikh, Klaus Hoeyer

**Affiliations:** Department of Public Health, Centre for Medical Science and Technology Studies, University of Copenhagen, Oester Farimagsgade 5, 1014 Copenhagen K, Denmark

**Keywords:** Denmark, Pakistan, Trust, Genetic research, Data sharing, Collaborative research

## Abstract

Trust features prominently in a number of policy documents that have been issued in recent years to facilitate data sharing and international collaboration in medical research. However, it often remains unclear what is meant by ‘trust’. By exploring a concrete international collaboration between Denmark and Pakistan, we develop a way of unpacking trust that shifts focus from what trust ‘is’ to what people invest in relationships and what references to trust do for them in these relationships. Based on interviews in both Pakistan and Denmark with people who provide blood samples and health data for the same laboratory, we find that when participants discuss trust they are trying to shape their relationship to researchers while simultaneously communicating important hopes, fears and expectations. The types of trust people talk about are never unconditional, but involve awareness of uncertainties and risks. There are different things at stake for people in different contexts, and therefore it is not the same to trust researchers in Pakistan as it is in Denmark, even when participants donate to the same laboratory. We conclude that casual references to ‘trust’ in policy documents risk glossing over important local differences and contribute to a de-politicization of basic inequalities in access to healthcare.

## Introduction

In recent years, a number of policy documents have emphasized trust as a crucial element in the promotion of data sharing and international collaboration in research (see, e.g., OECD 2014, 2016; OECD and GCOA 2015; European Commission 2016). In a decision reached by the European Union on May 27 2016 to work towards a paradigm of ‘Open Science’ (Council of the European Union 2016), it is written that a “trustworthy environment” for the use of samples is needed. Similarly, the Committee of Ministers of the Council of Europe adopted a recommendation (CM/Rec (2016)6) on research on biological materials of human origin emphasizing the importance of “earning trust and stressing the role of good and transparent governance of biological material of human origin stored for research purposes” (Council of Europe 2016: *preamble*). International platforms for research such as Public Population Project in Genomics and Society (P3G) also propose that “a key element of the success of a biobank is the trust and support of the public” (Wallace and Knoppers 2012). Concurrently, studies from multiple academic traditions have discussed trust in relation to participation in research using human biological material (Nicol et al. 2016; Lemke et al. 2010; Critchley et al. 2015; O’Doherty et al. 2011; Busby 2006; Platt et al. 2015; Cunningham-Burley 2006). Often one can get the impression that trust refers to a particular quality built into the relationships of collaboration as a sort of ‘factor’ with a particular effect on donations irrespective of context. But how do actual participants in collaborative international genetic research think about trust and the relationships in which they provide biomaterial and health data?

Important bioethical contributions have discussed the role of informed consent, privacy and confidentiality in relation to data sharing for health related research within a global framework (de Vries et al. 2011; Tassé et al. 2016), and empirical studies have sought to understand the interests of recruitment staff and researchers, highlighting the practical, social, legal and moral dimensions involved in transnational health research (Parker and Kingori 2016; Geissler 2013; Prainsack et al. 2016). In this study we focus on trust and take on the perspective of the individuals providing their blood samples and health data for research in a specific project. Based on interviews, we compare how donors in Pakistan and Denmark who deliver material to the same Danish laboratory conceive of ‘trust’ and the nature of their research participation. In Denmark the laboratory primarily collects material from people with balanced chromosomal rearrangements identified through public registers. In Pakistan, a national laboratory collects material from people with autosomal recessive disorders identified primarily through researchers’ personal contacts and snowball sampling. The material is then sent to Denmark. The differences between these two setups run deeper than that, however, and though we are easily lead to think of trust as a ‘factor’ that operates in the same way across geographical spaces when reading policies of international data sharing, we argue that ‘trust’ should not be thought of as the name of a phenomenon characterizing willingness to donate. People participate for much more complex reasons, and the ‘trust’ they invest takes many different forms. Broad policy references to ‘trust’ might divert attention from the hopes and concerns of research participants by ‘black-boxing’ what they *do* invest in the relationship with researchers and research projects when giving their genetic material and health data. We begin our argument by illustrating how ‘trust’ has been shown to have many meanings also in the literature.

### Academic discourses on trust

The concept of trust is used in multiple disciplines and with varying meanings. Some employ it as a descriptive and neutral concept; others use it as a prescriptive concept and ascribe positive values to it. Simpson (2012) suggests dividing the different understandings of trust according to three dimensions: affective, rational or normative. The affective dimension is described as an attitude of optimism towards the goodwill of others (Baier 1986). The rational understanding of trust involves seeing it as a forward-looking function of interest maximization (Coleman 1990). With a normative dimension scholars have focused on the moral expectation that others ought to do what is ‘right’, even in distant relationships (Hollis 1998). Holton (1994) bridges the normative and the affective dimensions when emphasizing the emotional aspect of expectations associated with trust: “When you trust someone to do something, you rely on them to do it, and you regard that reliance in a certain way: you have a readiness to feel betrayal should it be disappointed, and gratitude should it be upheld” (Holton 1994, p. 67). Some scholars have criticized the normative uses of ‘trust’, arguing that there is nothing inherently good or moral about trust (O’Neill 2002; Jones 2012). Instead of focusing on trust, they suggest assessing *trustworthiness* (Hardin 2002; Lehrer 2006; Aitken et al. 2016). Trustworthiness can be understood as ‘good reasons’ to trust. Misplaced trust, they argue, can be harmful, for example, when there is a discrepancy between the expectations of research participants and the interests served by the research institutions (O’Neill 2002). Hardin (2002) stresses that well-placed trust is present when the person or institution that is trusted is serving the interests of the one investing trust, terming this “encapsulated interests”. Acknowledging this conceptual diversity, Simpson concludes that trust does not refer to a single ‘phenomenon’: “Each way of thinking takes some relational situation as paradigmatic, and then builds an account of trust around it” (2012, p. 564).

Irrespective of Simpson’s critique, social and ethical studies of genetic research tend to refer to trust without conceptual clarification. Some scholars have scritunized the role of trust from an empirical bioethical perspective (De Vries and Kim 2008). Typically, “it” (whatever it is) is seen as related to the willingness to donate and described as vital to making research infrastructures work, essential for the effectiveness and success of biobanking research (Emerson et al. 2011). Beyond the field of biobanking, trust has been described more generally as a determinant for, or effect of ‘support to research’, because it builds relationships, provides legitimacy and reduces complexity (Gilson 2003; Bussey-Jones et al. 2010). This view of trust sees it basically as a means, rather than attributing a moral value to it (Sztompka 1999).

In a seminal analysis, Luhmann (1999) from a sociological perspective suggested studying trust in social systems by looking at what it *does*. He argued that trust is the name for a shortcut to social action that we need when we operate in contexts we cannot fully know or scrutinize. He suggests that trust and distrust basically do the same: they reduce social complexity that otherwise paralyzes our ability to act (Luhmann 1999). In practice, however, it is difficult to use this approach alone to empirically identify an object of study; trust is easily assumed to be there based on its functional characteristics. Furthermore, it ignores the affective dimensions also associated with trust by many observers.

So how do we study trust, if it is a concept with many meanings and not the name of a singular phenomenon? We propose to avoid searching for an understanding of what trust *really means*. Instead we use these aforementioned insights to encircle a vocabulary (affective, rational, normative) to understand relationships in their specificity, what people invest in them and why. We observe the relational qualities of ‘trust’, but instead of defining it with specific traits, or as reliant on particular ways of evaluating human qualities (cf. Humphrey 1997) we study how people operate in relations and use discourses of trust (in which the word acquires different meanings) to make sense of these relations. We thus approach trust descriptively rather than prescriptively. What we take from Luhmann (1999) is an inspiration to explore how trust becomes a *meaningful* ‘shortcut’ for the people who provide their material for research. We show the multifaceted way the term is *used*, and therefore question the uncritical use of terms like ‘trust-building’. Basically, we need to understand what is at stake for the research participants to unpack the concept of trust currently so popular and to reassess its ability to address their hopes and concerns.

### Methodology and settings

Because ‘trust’ is referred to in policy papers specifically aimed at promoting data sharing and international collaboration, we wanted to compare perceptions among research participants in very diverse contexts, yet who contribute material to the same research unit. Taking point of departure in one research laboratory in a high-income country, Denmark, and one of its partner-laboratories collecting material in a low-income country, Pakistan, we recruited research participants from each site following the practices and contacts of the respective laboratory undertaking the recruitment. We have followed the rules set by the Danish Data Protection Agency and a Research Ethics Committee in Pakistan. Qualitative research is not subject to research ethics committee approval in Denmark.

In Pakistan, Sheikh participated in the fieldtrips through which material (such as blood samples, biopsies, clinical tests and family history) is collected, and furthermore conducted 19 interviews in the homes of research participants with multiple family members. The interviews took place in December 2015 and January 2016 in urban and rural areas in and around three of the largest cities in Pakistan, while two were conducted over the phone. The interviews were primarily conducted in Urdu. Some conversations and seven interviews, however, were conducted partly in Punjabi (which Sheikh understands but does not speak fluently) with the assistance of a translator. Many of the families were living in poverty with limited access to health care, education and food, and had members suffering from severe diseases. The genetically affected families recruited for the laboratory’s research are identified through people known to the laboratory. To ensure variation, we interviewed families classified by the laboratory as belonging to ten disease categories, including different types of skin disorders, intellectual disabilities, recurrent pregnancy loss, abnormal growth, and other diseases known or imagined to have a genetic factor involved. All families agreed to participate.

In Denmark, the laboratory uses a national register of cytogenetic test results to identify people with balanced chromosomal rearrangements and invite them to participate in follow-up research. For some it has been so long since the tests were done that they have forgotten about them. When filling in a questionnaire for the genetic research project, the research participants were also asked whether they could be contacted by Hoeyer and interviewed about how they had experienced their research participation. Hoeyer conducted 23 interviews with individual research participants in a university office, over the phone or in the homes of the individuals, based on the wishes of the research participants (see also Hoeyer 2016 for further explication). An additional six research participants responded over email. The interviews were conducted in Danish. All quotes are translated by the authors, and all informants are given pseudonyms.

By participating in our study informants were in effect research participants in two projects—one dealing with genetics, and one dealing with their views and experiences. This duality has informed our analysis throughout, not least because many, when when we asked about their relationship to the genetic researchers, exemplified what they meant by talking about their relationship with us, the interviewers. For instance, Steffen, a Danish man, remarked: “[trust] means, well, now you have been allowed to record this conversation, and that’s fine (…), that’s trust for me,” Similarly Humaira, a Pakistani mother of a girl with microcephaly, who was a little disappointed about not having heard more from the genetic researchers, said that she was pleased to meet Sheikh because she gained new hope for her daughter’s treatment. In this way she used the interview situation to explain something about her relationship with the genetic researchers.

In our interviews in both Denmark and Pakistan we used the same guiding questions, and also coded the material according to the same three broad questions: How do research participants talk about trust and their research participation? In whom do they claim to invest trust and in which way? What would constitute a breach of trust for them? These three questions also provide the structure for the empirical analysis that follows.

### How do research participants talk about trust and their research participation?

In the following we illustrate how participants use trust to refer to expectations towards ‘something bigger’ than individual researchers, though in different ways in Pakistan and Denmark. Pakistanis tend to use a religious idiom and talk about their donation being in the hands of Allah, while Danes tend to use a secular idiom and refer to ‘the system’. Neither Danes nor Pakistanis talk about trust as infinite or unconditional: participants are aware of risks and that they depend on the care of researchers who could choose not to care for them. When people nevertheless participate in research they have their reasons, but the reasons vary and reflect different landscapes of opportunity, deprivation, hope and obligation.

#### Pakistan

In Urdu there are several options for translating trust, e.g., ‘itemaad’, ‘itminaan’ and ‘bharosa’. While itemaad and itminaan are words with close connotations to *reliability* and *being sure of something*, bharosa is often used in a religious sense. Bharosa was the most commonly used by research participants. Indeed, for most of them discourses of trust and faith are entirely interrelated. Khalida, for example, who was in her sixties and mother-in-law to one 18-year-old girl who had undergone several miscarried pregnancies explains trust in this way:

First of all we trust in Allah. We don’t have gold, we don’t have a lot of money, we’re just thankful that we are alive. So the first thing is that we can’t worry. You just have to trust in Allah, He will make things better. What will we gain from worrying? He brings a lot of disease, a lot of hardship. But he also brings a lot of joy.

To some extent, this religious framing reflects the precarious life circumstances in which people search for opportunities to invest hope, well knowing that they might be disappointed. Here, we need to acknowledge the level of precarity characterizing their daily lives, and how religious understandings shape understandings of disease and hope for a cure (Mattingly 2010). Several research participants talk about trust as believing in the good intentions of others, but then later, like Khalida, refer to Allah as mediating what will happen. This belief might be seen as partly reflecting what the literature describes as the affective dimension of trust (Baier 1986), though the secular philosophical vocabulary does not fully capture the way religion motivates the participants. Many use religious proverbs, especially one about the Prophet Muhammad having said that, “there is no disease that Allah has created, except that He also has created its remedy” (Sahih Al-Bukhari 7.582). To participate in research is one way of searching for that cure. Faisal, a 26-year-old man totally invalidated from a severe case of myopathy thus states how he has high expectations of the research due to his trust in Allah: “He is the one who can cure any disease”. These participants thus talk about trust, but they do not invest it directly in researchers. Rather, it is a religiously mediated trust.

Nadeem, the father of two girls whose disease had a late onset, in contrast, expressed great confidence in science per se: “Science has made such great success, anything can happen. Science has come very far!” We should note, however, that this man had been sending his daughters to *Peers* for many years. *Peer* is originally the Urdu title of a spiritual guide, but has developed into a type of business where some men provide ‘religious services and advice’ for example on how to drive out what they identify as evil spirits entering the body. Nadeem’s statement should therefore not be seen as an unconditional devotion to science, but more as an indication of what was general for most of the Pakistani informants: their willingness to embrace anything that can support their hope for a cure for their children. Umar and Humaria, the parents of a girl suffering from a mild case of microcephaly, where her memory is quite affected even though she had a high level of self-care and awareness, explained that they were happy that researchers came to their village because it gave them hope. Humaira described the visit in these terms: “We were glad they took the blood (…) they come because a treatment is possible, that is obvious. Why would they come if there is no treatment?” Here trust is spoken of as embracing hope in situations where there is little else to provide it. This type of trust can be seen as an investment conditioned on deprived opportunities. According to McGeer (2008), trust and hope are intimately interconnected: our capacity to hope supports our capacity to trust (McGeer 2008: 237). Considering how unlikely it is that this research will involve a cure for the children involved, discourses of hope and trust involve a dilemma. If the trust is based on hope for something that will never come true, is it then similar to what philosophers call ‘misplaced trust’ (Hardin 2004) or what Appelbaum (Appelbaum et al. 1987) famously tagged ‘a therapeutic misconception’? Or does this portray a case of ‘undue influence’ (Lavery et al. 2007)? Rather than employing these bioethical categories to define a particular breach by context-free international standards, we suggest a need to appreciate the specificity of how the terms of enrollment engage the ‘trusting’ parties’ sense of opportunity, deprivation and hope.

Other participants stated that trust was not important for their choice to donate. Hussain, a man in his forties, thus explained: “Giving the sample has nothing to do with trust. If they do anything for our betterment, then we have trust.” For Hussain, trust is something to be earned in a way that combines rational and normative dimensions. Some combined this understanding of trust with a religious framing. A woman stated for example that “if they are doing good things, thank you, may God bring good things for them. If they have any other intention, then God knows better.” Some donors thus describe their willingness to donate as reflecting neither force nor trust. Rather, research participation is a ‘gamble’, an insecure act involving risks.

#### Denmark

The Danish word for trust is ‘tillid’ and it has many of the same connotations as the English term, though it is rarely used in any religious sense. For example, you would not say ‘tillid til Gud’ as in the English expression ‘to trust in God’ or as in the Urdu term ‘bharosa’. Furthermore, Denmark is known as a secular society with few references to religion in public discourse (Zuckerman 2008). In Denmark, research participants often use an idiom of ‘trusting the system’, which, though entirely secular, in some ways resembles the way Pakistani research participants talk about trusting Allah. Christian, a man in his sixties thus explains how he has “trust in the system that is for sure” and adds how this is his main reason for participating in genetic research and in our interview: “That is why I have trust. That is why I am sitting here. Because I *am* a citizen in a proper little country where we behave in proper ways.” Similarly, Jens Ole said: “The doctors want what is good. And I trust the doctors. I trust science. I have full trust in science.”^1^ Anne-Sophie says “I guess I basically have faith in the system, or whatever you call it” and Thomas similarly says “I trust them to guard [personal information] as they should. There are rules for these things in Denmark”. Both Anne-Sophie and Thomas also remark that they do not know the specific rules. Instead it is as if they build on life-long experiences with the services of a welfare state. They see their relationship with their country as involving a sense of mutual obligation: for them to participate and for the ‘system’ to care for them, but the actual uses of their samples remain abstract to them and to most of the other participants: they are just used for ‘research’.

Importantly, however, this seeming sense of confidence in a functional ‘system’ is not unconditional. In Denmark, every biomedical research project involving the use of samples is registered and assessments are made by ethical committees. When Ulla, the mother of a disabled child, talks about these ethical committees she says that she expects responsible persons to take care of her interests, but then continues: “But still… I can’t help doubting, right? (...) No, I have to trust that the people working with these things will do it in ... in the right way. And that it is to enrich our society and not to mess anything up.” This awareness that they cannot know if researchers will take care of them is a particular type of uncertainty that is constitutive for the type of trust Ulla is describing. It would be mistaken to think of trust as a substantial belief in others (Gross 2012, p. 431). Several research participants mentioned cases of data leakages on public figures, including a famous leak of information about the sexuality of the prime minister’s husband. Several participants who mentioned such leaks, later remarked that they themselves were probably not interesting enough for any media to broadcast their data. A sense of doubt and awareness of risk is also mentioned by Lisbeth, but for her trust is a choice with a greater sense of emotional and affective implications (Holton 1994): “What is trust? Well it is that you want to stay in another person’s care, and that you trust that they won’t hurt you... or harm you, or do life-destructive, offensive things. So it’s a big thing of course to trust someone else.” Note how she too describes trust as a *choice* that involves a risk. Risks must be accepted, however, if science is to have any chance of progress, as several participants remarked. Unlike the Pakistani participants, most of the Danes thus invest in hope for future generations rather than expecting alleviation for their own hardship. Cutting across these ideas is not only awareness of the involved risks, however, but also a sense of trust being dependent on belief in something similar to what Hardin (2004) called ‘encapsulated interests’; namely that the ‘system’ in most cases strives towards the same aims of scientific progress as the participants.

Both the way research participants talk about trust, and their research participation in Pakistan and Denmark, are thus conditioned on very different experiences and life circumstances. In both countries, people engage in a relationship knowing that they can be let down. What they invest is mediated either by divinity (in Pakistan) or by ‘the system’ (in Denmark). Nevertheless, it would be erroneous to equate the emotional stakes involved in Denmark and Pakistan, and it would be to misconstrue the reasons for research participation if we said that people participate *because* they ‘trust’ researchers. In order to understand further how people construe the relationships they enter through research participation, we now go on to our second question: In whom do they invest (what they personally conceive of as) trust?

### In whom do research participants claim to invest trust, and how?

In both countries we asked research participants to elaborate on who they themselves, as participants, trusted. We did so to understand how they assessed the relationships they encountered through research, and where they would accept their samples being sent. In Denmark, a pattern emerged which can be described as a set of concentric circles of declining trust, from the health professionals they know, to the national health services, to the European Union and finally the rest of the world. In Pakistan, in contrast, people talk about trust as invested in those closest and furthest away, while mistrust is expressed towards the national level. In the following we suggest that this could reflect ideas about who might potentially help them. The difference between Denmark and Pakistan again reveals significant aspects of the contextual nature of the relationships in which donations are made.

#### Pakistan

With no genetic registers, Pakistani researchers have to identify genetic carriers through personal networks. In a society with inadequate access to healthcare, most people also navigate disease with the help of networks and the local community. Patients and their relatives consult family, friends, neighbours, doctors or someone they meet in mosques or in other contexts where they feel secure. Research participants described how they felt obligated towards their family to take all the advice they can, both from biomedical health professionals and other realms of care. The research project enters their lives through this network model when contacts are established between named researchers and the suffering families through mutual acquaintances. Khadija, an elderly woman who could not read or write, explained why she thought well of the researchers:

The people who came are literate and well read. They were doctors. I tell you, with very good manners, with gloves, in a van, they also checked the height of my children. (…) It was hot that day so we got bottles [soft drink]. In a good manner they checked us, looked at us before they left.

Most research participants refer to researchers as ‘doctors’, even though some of them were just finishing their graduate research and not even in medicine. They are, however, typically seen as possessing authority and kindness, which are characteristics that research participants were looking for in their strategy to find a cure for their family members.

In contrast, several of the research participants expressed negative feelings when talking about the national health system. One father of six boys with myopathy, out of which three had passed away, expressed his anger with the government of Pakistan for not having helped him. Through digital media, television and 3000 flyers he had tried to seek financial help for medical care, and was devastated by the fact that the politicians had not reacted. Patients, or their families, have to pay for the services of healthcare professionals, who sometimes are seen as more focused on the money than health: “In order to run their own shop they will keep you coming over and over again,” as he said.

The state of affairs in Pakistan was also a concern of a young man studying for his MBA and taking care of his siblings while his parents were abroad:

Here in Pakistan people will shoot you in the fear of polio. It is the reality. There are other even worse realities I can’t discuss. (…) And that is why Pakistan is lacking behind in regards to polio. (…) I would also be hesitant to agree to a research team coming here. But this project was recommended [by close friends].

Here this educated man is appalled by the national situation and turns to local networks (friends and family) in search of a way of maneuvering. As the Pakistani researchers are employed by a national government institution, it can be seen as paradoxical that the participants can think so badly of national institutions yet still embrace the work of the genetic researchers. However, by entering through social networks, the researchers are seen as more local and therefore not in the same category as the ‘national level’.

Following the general perception that a fake hepatitis vaccination campaign was carried out in which DNA samples were collected to help the United States track Osama bin Laden in 2011, other foreign agencies collecting samples in Pakistan for research purposes (also when unrelated to vaccinations) became surrounded with anxiety and sometimes even seen as a potential threat of terror in their own right. Hamza, an invalidated 23-year-old male, had also been worried about giving the sample because of the risk of terrorist acts: “A lot of people are into anything for the money. And then they go into terrorist acts.” He elaborated that: “The needle they use, you know... There could have been poison in it. But no, we, it didn’t happen, not that.” Despite the anxiety and spill over of sceptisism from the vaccinations campaigns to collections of biomaterial for research, most families were noticeably happy that their samples were being sent abroad for research and enthusiastically agreed to provide samples to researchers. The typical reply was, “Absolutely you can send the samples. We don’t mind at all.” How can this be?

We propose that the participants express trust in international partners because these partners represent resources not otherwise available. Research participants were told that laboratories in Pakistan have neither the money nor the technology to properly analyze the samples: “It costs 2.2 lacks [2102 USD] for one test. So people from abroad say that they can do this for us,” Sameena explained. In this way, ‘abroad’ becomes a place of opportunity. They are looking for what maximizes their chances for treatment and cure, similar to what Coleman (1990) calls the rational dimension of trust. In most cases research participants were not aware about the information from to the consent sheet that researchers are supposed to provide verbally about the use of samples, but when they did remember something, it was the collaboration with international partners. Articulations of trust seem to reflect people’s perception of opportunities for help more so than processing of information about and awareness of procedures and use. We thus find that the specific information research participants want and remember is the information that they can use when dealing with the challenges of their daily lives. We should not assume that people invest ‘trust’ in foreign researchers in any general sense, as if they were affectively connected to them. Rather, they trust foreign researchers to have an ability to do something, and hope this ability will be used in their favor.

Several families were under the impression that, through the international partner, their children might be taken to Denmark, Germany or Sweden for diagnostics, treatment or drug development. Binish, the mother of three small children suffering from Charcot-Marie-Tooth disease for example said: “They said that they were going to do some tests in Germany, I mean test the blood over there. Then they would try to take our kids over there for treatment.” Several others also explained that the researchers collecting samples had mentioned this as a possibility, if a treatment was found for the children. Some had the impression that the ones collecting the samples would cover the charges for the treatment. When people want to send their children abroad, it indicates their level of desperation. This should not be glossed over as a matter of ‘trust’, as if ‘trust’ could explain their research participation: they participate to get help. They are trying to get access to resources and possibilities for care for themselves or their loved ones (see also, Whyte et al. 2013; Bruun 2016).

#### Denmark

Whereas the Pakistani research participants like the idea that their samples are being sent abroad, Danish research participants typically invested less and less trust the further away their samples and health data would go. Most preferred research being done within Denmark, but had in fact thought very little about what had happened to their sample. Maria explained: “My immediate thought is that Denmark will protect me the most [long pause] but I have no clue. I haven’t given it a thought at all.” Maria thinks that everyone in Denmark should have their samples and fingerprints recorded, and that all health records should be available for research in digitized format. She is confident that the tax-financed National Health Service will make good use of her health data. Steffen is also comfortable with the way the samples are treated within the Danish system, and referred to the ‘high standards’ in Denmark compared to other EU countries. However, one informant, Lisbeth, a woman working in governmental research herself, was skeptical about the organization of the Danish research system as she found it characterized by an overly lenient use of samples.

Primarily skepticism was articulated when Hoeyer asked the Danish participants what they thought about their samples being sent to, for example, China, in case whole-genome sequencing would be cheaper there (the world’s supposedly biggest genome sequencing company is situated in Beijing and has opened a branch in Denmark). Steffen, among others, thought that the risk of abuse of his samples would be greater in China, or in other forms of cross-national collaboration. Christian stated that China is “too far away.” Anette did not mind samples being sent to China, and would leave it up to researchers. She assumed ‘the system’ would know what is reasonable.

The distinction between public and private research was another way of discussing in whom participants invest trust. Some remarked that if their samples were to be sent to private companies, they at least would like to be asked first (while accepting presumed consent for public uses). Some believed that private companies from Denmark were “Okay”, as some participants said, but not foreign companies; others saw all companies as less obliged to care for interests other than financial profit. In this way, Danish research participants invest trust according to a set of concentric circles: the closer to the donor, the more trustworthy; and relative distance can be institutional (public/private) or geographical (Denmark/EU/China). Moving further away in the circles involves risks, but risk-taking can be legitimate if something is to be gained: for example, if it is economically efficient or the right competences are elsewhere. Anne-Sophie believes the samples should be analyzed where it is cheapest: “I think, that [if it is cheaper], they should do what makes sense. Logical sense.” Kirsten looks at competencies and thinks that China could be fine, because they are “good at what they do.” Hence, in some ways the Danes invest trust according to some of the same parameters as the Pakistani research participants, that is, ability to achieve aims, but under structurally different conditions and with a focus on risk, rather than an emphasis on hope. The ‘trust’ they talk about does not refer to the ‘same’ phenomenon. This point becomes even clearer as we now turn to what research participants would consider potential *breaches* of trust.

### What would constitute a breach of trust?

Overall, reflections on breaches of trust in Pakistan were tied to families’ quest for health, while in Denmark breaches of trust were related primarily to disclosure of sensitive information or unethical research applications. Research participants obviously have different things at stake in the two countries. In both places, research participants appeared somewhat uncomfortable when asked to speak about potential breaches in trust. Their reflections on breaches of trust give us insights into not only what they understand by trust, but also what talking about trust does to and for them.

#### Pakistan

When Sheikh asked about breaches of trust, Pakistani research participants reacted either with skepticism or categorical denial—or they used the question as an opportunity to state their dissatisfaction with researchers. One donor, Kauser, responded harshly: “What are you implying?” Another donor simply ignored the question, even when asked a second and third time. These reactions exemplify how discussion of trust can indicate suspicion and interfere with the relationship as such, and not just describe it (Baier 1986, p. 260). Some categorically stated that nothing could undermine their trust in the researchers. In contrast to the doubts they had just expressed when talking about what trust meant to them, these donors now denied having any worries. One father, Nadeem, answered our question saying: “There is nothing, we will cooperate, as long as you help our kids, we don’t need anything besides that.” He did not wish to talk about breaches of trust, because he did not wish to endanger the possibility of receiving help from the researchers. Humaira similarly promised that if her daughter was given treatment, “we will be cooperative, all the way”. These reactions show how questions about breaches are converted into potential tests of a relationship they still depend on. In this way, talking about trust can be a way of ‘doing’ relationships in particular ways.

Another group of research participants used the topic to air their dissatisfaction. For them breaches of trust were not hypothetical. Most of these participants had provided their samples more than 2 years before Sheikh interviewed them. For them the lack of contact from researchers after having provided the sample was considered a breach of trust. Maida, a female donor with recurrent pregnancy loss was dismayed because she had expected researchers to tell her the reason why she had had three miscarriages. Her main reason for donating her fetus and blood sample to research was to understand why “her baby wasn’t growing” and she explicitly said that she would never donate her fetus again, even if it was for the “betterment of others.” Many framed lacking response from the researchers as betrayal: “They said they would stay in touch, but they didn’t contact us at all” and “they just took the blood and left, even though they said they would come back.” This clearly depicts a discrepancy between the expectations of research participants and the work and possibilities of the research institutions. Indeed, most researchers in Pakistan want to help the families, change consanguineous patterns and benefit society. They too have hopes and concerns, but they are just like the research participants, limited by their resources.

#### Denmark

The Danish research participants had not given breaches of trust much thought prior to the interviews. When asked, many focused on data leakage and confidentiality issues, as already mentioned above. Others said that if their sample was used for something other than what the researchers had said they would use it for, it would challenge their trust. Interestingly, they could not remember what they had agreed to in the first place. Some people relate ‘breaches of trust’ to unethical research purposes. However, what is seen as ‘unethical’ differs among the research participants. Lisbeth explained that it is not legitimate to enhance knowledge just for the sake of getting to know more. It has to promote health and wellbeing. Niklas said it would be a breach of trust if biological weapons were to be developed using his sample, while Morten mentioned cloning as a potential breach of trust, and Steffen mentioned bodily enhancement, such as muscle increase. None of the concrete examples are relevant for the research conducted by the laboratory, and for the research participants they probably sprung to mind only as a consequence of being asked an unexpected question. Other research participants were concerned about research that no longer focused on health, but things like identifying criminal genes, genes for sexuality, fraud and so on. Research that would only focus on commercial gain was also mentioned. So whereas breaches of trust in Pakistan can be a concrete experience and relate to people’s struggle for health, contemplation of breaches of trust are in Denmark more hypothetical and related to privacy and respect for personal values.

## Conclusion

In this paper we have problematized the tendency in policy documents to refer to trust as a particular ‘factor’ with a given effect on donations irrespective of context. Just as the literature contains many different concepts of trust, our analysis illustrates how references to ‘trust’ can do many different things for research participants in different settings. Our analysis indicates that there are variations in how research participants conceive of trust both between and within Pakistan and Denmark (Table 1). We do not wish to generalize these findings beyond the particular collaboration that we studied, on the contrary, we wish to emphasize that these differences and variations should not be read as reflections of different ‘cultures’ or static modes of trusting. In different contexts and situations, people have different hopes and concerns, different opportunities and different cosmologies. When participants discuss trust they are trying to shape their relationship(s) with researchers while simultaneously communicating important hopes and fears in light of their situation. The generalization we propose is therefore not one about what trust means in Pakistan or Denmark, but one about how to approach the issue of trust: we should avoid thinking of trust as a factor with a particular meaning and effect, and instead explore what people invest in research collaborations and why.

**Table 1:**
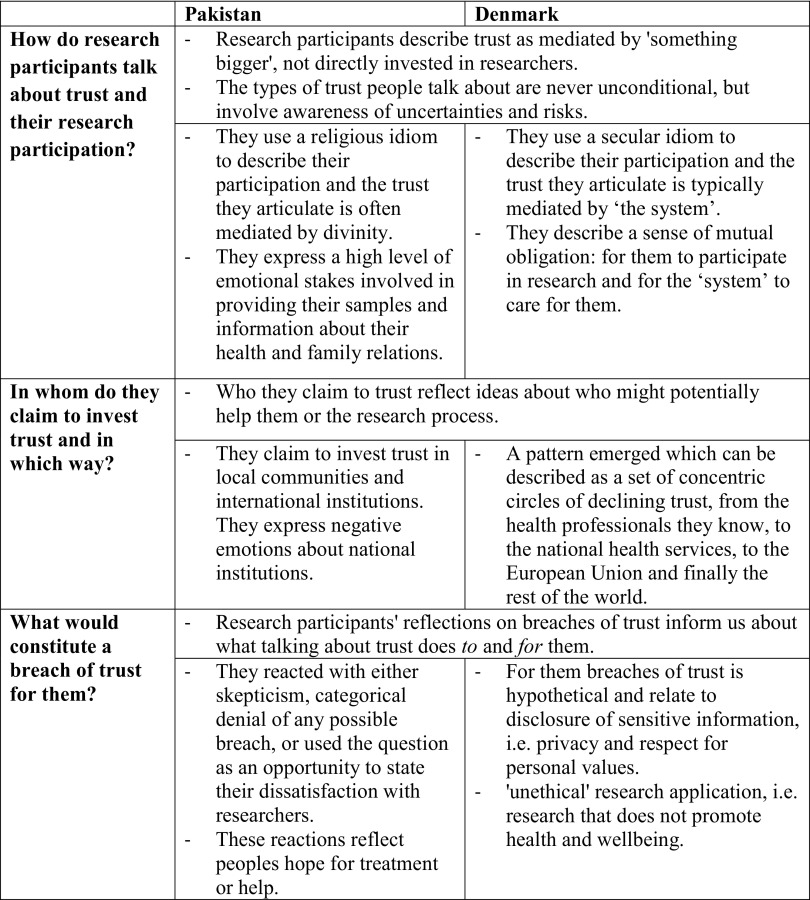
Summary of key findings comparing dominant perceptions of trust in Pakistan and Denmark with regard to three key aspects

The findings are separated into what they share and how they differ. It is important not to essentialize these similarities and differences, but instead use the awareness of potential differences to explore what trust means and does for people in actual processes of recruitment in these and other countries

We could use our observations of divergent meanings of trust to dismiss the concept altogether, but then we would also limit our understanding of the concerns that people seek to communicate with it. Instead, we suggest opting for a more nuanced conceptual understanding of trust. Our point is to show that variations in conception are determined by how the term is used, i.e. what trust *does* for these people, and thus highlight the contextual nature of the relationships in which donations are made and what references to trust do for them in these relations.

There are many reasons why policies of data sharing and international collaboration focus on harmonization. Simple and harmonized rules make collaboration easy. However, when harmonization does not take into account local interests, it risks jeopardizing the sustainability of the very type of research the policies were produced to promote. Casual references to ‘trust’ in policy documents gloss over these significant differences, and thereby risk de-politicizing basic inequalities in access to healthcare. Glib policy statements about ‘trust’ can end up legitimizing use of people’s donations (following an implicit argument of the type “we can use this biomaterial because we would not have it if people did not trust us”), without paying adequate attention to the actual reasons people have for participating in research.

In Pakistan, unfulfilled expectations today pose a problem for future sampling, as seen when disappointed research participants refuse to take part in resampling. The current way of collecting samples and considering trust in instrumental terms thus constitutes a problem for both moral and practical reasons. It has been acknowledged as such by the laboratory in Pakistan, but it takes international recognition to change the conditions under which they collect material. In Denmark, similarly, researchers take great care to follow up on donor concerns, but most of this work is considered irrelevant by funding agencies and is done by dedicated researchers as an extra activity (Hoeyer et al. 2017). In Pakistan, most of the work researchers would like to do to help participants also remains unfunded. Data collectors play an important role in shaping legitimate practices when interacting with research participants and community members (Kingori 2013). Harmonization and easy references to trust must not become a reason for disregarding what is actually at stake for participants in their local context. In many low-income contexts, people do not donate *because* of trust; they need to trust to donate; and they need to donate to opt for help. The sustainability of future international research infrastructures depends on the ability of research institutions to understand how they affect the lives of the research participants, and explorations of local understandings of trust can be a good place to start.

## Footnotes

1 Interestingly, there is concurrently an ongoing discussion about a ‘crisis of trust’ in Denmark, and a recent report indicates increasingly lower levels of ‘trust’ in the healthcare system (Mandag Morgen and TrygFonden 2016). However, this is not reflected in our material.
